# Two-staged posterior osteotomy surgery in complex and rigid congenital scoliosis in younger than 10 years old children

**DOI:** 10.1186/s12891-021-04682-y

**Published:** 2021-09-13

**Authors:** Sheng Zhao, Xuhong Xue, Kai Li, Feng Miao, Bin Zhao

**Affiliations:** Department of Orthopedics, The Second Hospital of Shanxi Medical University, No. 382 Wuyi Road, Shanxi 030001 Taiyuan, P.R. China

**Keywords:** congenital scoliosis, rib deformity, hemivertebrae, Y-shaped osteotomy, two-staged surgery

## Abstract

**Background:**

Congenital scoliosis caused by failure of multiply vertebral segmentation with concave fused rib or unsegmented bar combined with contralateralhemivertebra is usually rigid and produces enormous asymmetric growth. Fusionless techniques have less advantage and come with some complications. Paucity of data was reported for children with complex congenital scoliosis using two-staged osteotomy surgery.

**Methods:**

From 2006 to 2016, 11 patients less than 10 years old undergoing two staged osteotomy surgery for complex rigid congenital scoliosis were retrospectively reviewed. The analysis included age at initial surgery, second surgery and at the latest follow-up, and complications. Changes in coronal major curve, thoracic kyphosis, lumbar lodorsis, apex vertebra translation, T1-T12 length, T1-S1 length, trunk shift, and SVAwere included in radiological evaluation.

**Results:**

In all, the mean follow-up was 72.5 ± 23.8 (42 to 112) months. The mean flexibility of the spine was 17.4 and 17.8 % before two surgeries. The mean age at the initial surgery was 6.6 ± 2.6 (2.5–10) years. The mean fusion level was 4.6 ± 1.3 (2 to 6) segments. The mean scoliosis improved from 67.4° to 23.7° after initial surgery and was 17.4° at the latest follow-up. The average increase of T1-S1 length was 0.92 cm per year. No patients had neurological complications.

**Conclusions:**

Two-staged osteotomy surgery including hemivertebrae resection or Y-shaped osteotomy can achieve good radiological and clinical outcomes without severe complications. This procedure can be an option of treatment for complex congenital scoliosis.

## Background

Children less than 10 years of age was defined as early onset scoliosis (EOS). The management of congenital scoliosis (CS) patients of EOS poses significant challenges to the spine surgeon. Progressive of curvature and thoracic cage deformity could be unavoidable if left untreated or inappropriate treatment. Most of them may suffer from pulmonary hypoplasia and thoracic insufficiency syndrome [1, 2]. Multi-segment spinal fusion surgery is not appropriate in these cases considering immature of bone. Prior studies suggested spinal fusion at younger age was correlated with decreased pulmonary functions [3]. In addition, more fused thoracic segments were associated with lower pulmonary function [4]. For patients with maximum growth potential, fusionless techniques not only can permit spine growth, but also maintain acceptable correction. Unfortunately, repeated surgeries are needed every 6 to 9 monthsto allow the spine and thoracic cage to grow [5, 6]. And the implant-related complications, wound infection, increased costs due to multiplerepeated procedures, and psychological consequences are of great concern[7–9].

For these patients with complex congenital scoliosis due to unilateral failure of multiple vertebral segmentation, unsegmented bar combined with contralateral hemivertebra, or vertebrae anomalies with concave fused rib, their curves are usually rigid, and growth potential is restricted to some extent. Growth friendly techniques including growing rods have less advantage and come with some complications [9]. In addition, most of them have intraspinal anomalies. These anomalies are believed to limit the movement of the spinal cordand thus increase the risk ofneurological complications inprimary or repeated lengthening surgeries [10]. Under this circumstance, we have developed two-staged posterior osteotomy surgery in order to reduce patients’ burden and the risk of complications. The concept of this technique is to achieve partial curve correction by removing the primary driving force of spine deformity in apex vertebra region. Meanwhile, fusion level is required as short as possible to allow continuing growth of the non-fusion spinal length. The strategy of correction is based on the gradually improvement from large curve to moderate, and then mild or even normal.

From this, we propose the following hypothesis: two-staged osteotomy operation including hemivertebrae resection or Y-shaped osteotomy can achieve good radiological and clinical outcomes. Currently, we apply this method to treat complex and rigid CS with rib deformity. The aim of present study is to evaluate the radiographic and clinical outcomes of the two-staged osteotomy surgery in patients less than 10 years old.

## Methods

From 2006 to 2016, all patients with congenital scoliosis less than 10 years of age were identified from our database. Among of these patients with rigid scoliosis due to unilateral failure of multiple vertebral segmentation, unsegmented bar combined with contralateral hemivertebra, or vertebra anomalies with concave fused rib have undergone two-staged osteotomy surgery. In all, 11 consecutive patients were included with mean follow-up of six years.

Preoperative evaluation included meticulous neurologic examination, full length posteroanterior and lateral radiographs. 3-dimensional CT scan in entire spine was performed to detect details of vertebra and rib anomalies. And magnetic resonance imaging was performed for detecting intraspinal abnormalities. Cardiovascular and urogenital examinations were performed to detect congenital heart diseases and abnormality of the renal system [11]. None of the patients had neurologic abnormalities. Initial surgery was indicated by proved or expected deterioration of the deformity, less response to non-operative treatment.

All radiological measurements were calibrated and corrected for magnification to represent actual change by an independent observer. These data were based on free standing posteroanterior and lateral radiographs taken before surgery, after surgery and at the final follow-up. Full spine radiographs were reviewed to record the location and number of the abnormal vertebra, segmented status, coronal major curve, apex vertebra translation, trunk shift, T1-12 length and T1-S1 length in coronal plane; thoracic kyphosis, lumbar lordosis and SVA in sagittal plane. The trunk shift (TS) was evaluated as the distance between a vertical line drawn from the middle of C7 body to the middle of sacrum. T1-12 length was the distance from T1 to lower endplate of T12. T1-S1 length was the distance from T1 to upper endplate of S1. Sagittal alignment was measured as the distance between C7 plumb line and the posterior superior corner of S1. Flexibility was used to estimate the rigidity of the curve. The curve was considered stiffness when it was less than 30 %.

Demographic data were recorded including sex, age, height, Risser sign and triangular cartilage. Data were recorded regarding the age at the time of surgery, the levels fused, the type and level of instrumentation, and complications.

### Surgical techniques

**Fig. 1:**
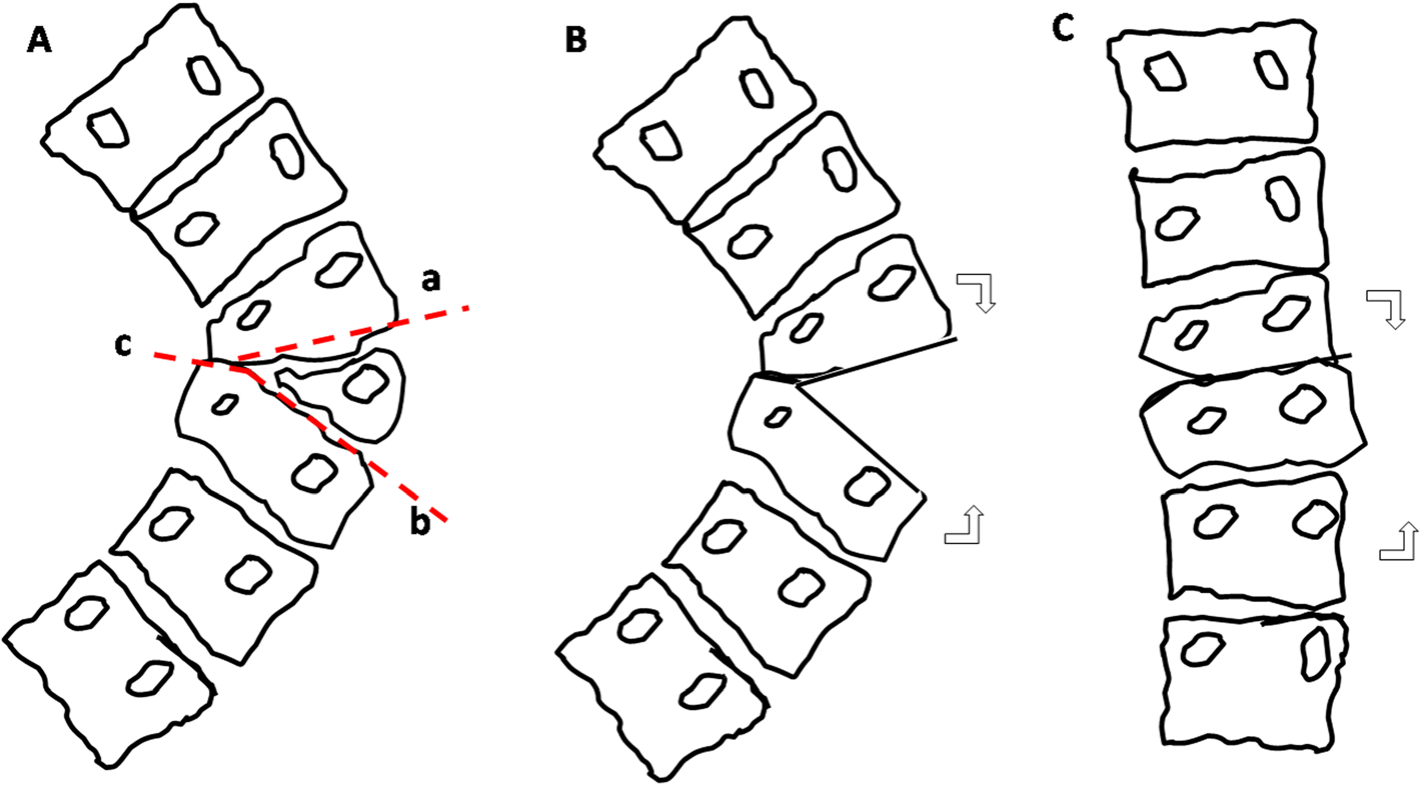
Schematic figure of Y-shaped osteotomy in coronal plane: the apex vertebra was selected as center of osteotomy (**A**). Lamina, superior and inferior articular process, and pedicle, as well as 3/4 vertebral body on the convex side were removed (**B**). After gradually compression, convexity closing, concavity opening and a quarter of concave side preserving as the hinge, forming an inverted Y-shape (**C**)

**Fig. 2:**
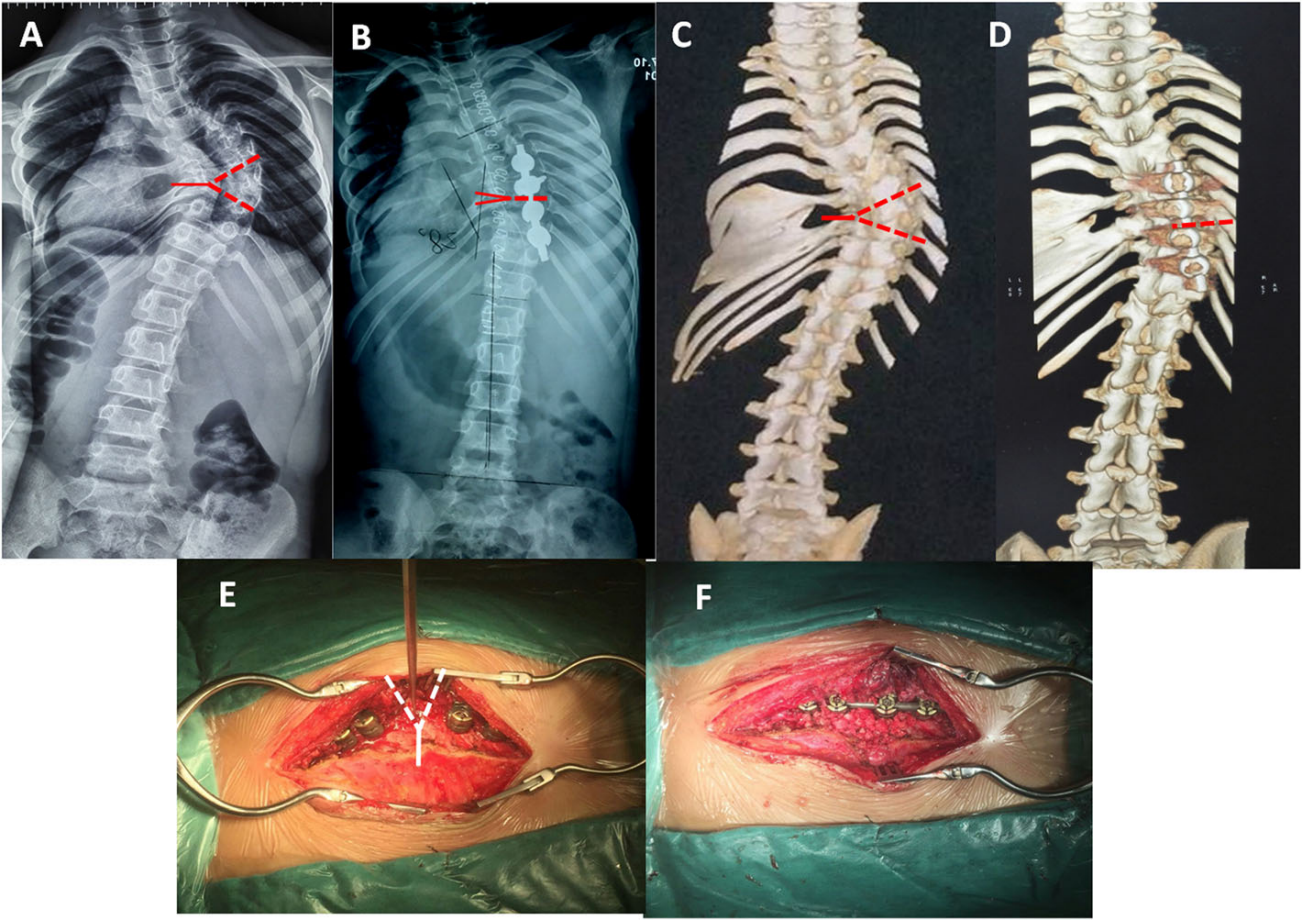
Pre- and Post operative photographs of a 10 year old patient who presented with complex congenital scoliosis; 3 dimensional CT showed failure of T5-9 anomaly vertebrae segmentation and massive fused rib; T8 Y-shaped osteotomy was performed with unilateral fusion (**A,B,C,D**). Interoperation image: convex side only to expose and unilateral pedicle screws were inserted. Gradual compression until the gap was closed after osteotomy. Bones graft for fusion of the posterior elements(**E,F**)

The procedure of hemivertebra resection (HVR) was performed according to our previous report [11, 12]. The concept of Y-shaped osteotomy was characterized by controlled convexity closing, concavity opening and a quarter of concave side preserving as the hinge (Fig. 1 A). Briefly, the patient was positioned prone on the operation table. The abdomen was left free to reduce intra-operative epidural bleeding. A standard midline skin incision and subperiosteal dissection was performed to expose osteotomy site and adjacent vertebrae to be fused. Unilateral pedicle screws were placed after taping in 1–2 levels above and below the osteotomy site using free-hand technique (Fig. 2). Fluoroscopy was used to confirm the osteotomy site. Single vertebra in apex region can be selected as the center of osteotomy. Lamina, superior and inferior articular process, and posterior part of the pedicle were removed. The remnants of the 3/4 vertebral body on the convex side were removed using osteotome, rongeur and curette until the bleeding bone was reached. Concave quarter vertebra was cut off and retained with a chisel. In patients with contralateral bar and rib synostosis, the bar was cut and the synostosed rib heads were removed (Fig. 1B). Epidural veins were cauterized by bipolar cautery to allow clear visualization. Subsequently, V or Y-shaped space were formed, pre-contoured rod was connected to the screws. Gradual compression was applied until the gap was closed [11]. On this moment, concave residue vertebra, rib head and soft tissue were slight opened, which formed an inverted Y-shape ideally (Fig. 1 C). Caution must be taken to make sure the exiting nerve roots and the dura not impinged. Bones retrieved during osteotomy were used as graft material for fusion of the posterior elements (Fig. 2).

Some further curve progression may necessitate additional surgery especially during the adolescent growth spurt. The indications of second surgery included significant curve progression in coronal and sagittal plane, severe proximal or distal junctional kyphosis and pedicle screw loosing or cutting. The fusion level was as short as possible, which depends on both severity of spine deformity and flexibility of curve. In general, one or two vertebrae above and below were fused after hemivertebra resection. However, for severe and rigid spinal deformity, more fusion level may be needed.

After surgery, all patients were fitted with a brace to protect the instrumentation for three months. All patients were followed up regularly.

### Statistics

Pair t-test were used to analyze the difference of these radiographic parameters, including coronal major curve, thoracic kyphosis, lumbar lodorsis, AVT, T1-T12 length, T1-S1 length, TS and SVA at pre-operation, post-operation and at the final follow-up. SPSS version 24.0 (SPSS Inc., Chicago, IL) was used in all statistical analyses. *P* value less than 0.05 were considered as statistically significant.

## Results

Of the 11 patients in the study, 7 were female and 4 male. Ten patients had hemivertebra, including 4 full segmented hemivertebra and 6 semi-segmented hemivertebra. According to the Winter’s classification, three cases were type 1 and eight cases were type 3. There were 6 cases with concave unsegmented bar, 2 cases with bloc vertebra, and 7 cases with complex rib anomalies. Two cases were associated with split spinal cord malformation, presenting as both bony and fiber septum. There was one case with synringomyelia and three cases with tethered cord. The demographic and surgical features were described in Table 1.

**Table.1:** Demographic data, deformity and surgical features in two staged surgery for complex congenital scoliosis

Patients	Age	Sex	Spine abnormalities	Associated with abnormalities	Follow-up(months)		Surgery		Fusion level		Correction of major curve (°)		Correction rate	
					1st	2nd	1st	2nd	1st	2nd	1st	2nd	1st	2nd
1	8	M	T5 HV(SS);T2-4 bloc vertebra	2-3rd,5-6th fused rib in concave side; Sprengel deformity	11	101	T5 HVR	T2 Y-shaped osteotomy	T4-7	T1-3	78 − 16	40 − 5	79.5 %	87.5 %
2	5	M	T8 HV(SS)with contralateral bar; *FS*: T7-10	8-10th fused rib; 9th bifid rib	57	37	T8 Y-shaped osteotomy	T9 Y-shaped osteotomy	T6-11	T6-12	49 − 18	48 − 14	63.3 %	70.8 %
3	4	F	T6 HV(SS); *FS*:T5-9		28	52	T6 HVR	T7 Y-shaped osteotomy	T5-7	T5-10	50 − 24	40 − 10	78.5 %	75.0 %
4	9	F	T7-8, T10-12bloc vertebra; BFV:T8,11	7-8th fused rib in concave side SCM: type I and II; Low conus:L3	36	18	T9 Y-shaped osteotomy	T10 Y-shaped osteotomy	T7-11	T7-L2	65 − 14	42 − 10	58.2 %	76.2 %
5	4	F	T10,12 HV(FS)	T11-L5 bifid spine; 9th bifid rib in concave side	36	24	T12 HVR,T10 hemivertebrapartial resection	T11 Y-shaped osteotomy	T8-L3	T8-L3	91 − 30	51 − 26	60.4 %	49.0 %
6	8	F	T5/6 HV(SS); WV:T5,6;Lamina fused:T3-4, T6-8	4th bifid rib in concavity; 5-6th fused ribs in convexity; Low conus(L5)	41	18	T5/6 HVR	T6 Y-shaped osteotomy	T4-8	T3-9	48 − 26	46 − 18	46.5 %	60.9 %
7	10	F	T5,9 HV(FS)		24	36	T5,9 HVR	T4,8 Y-shaped osteotomy	T4-6,T8-10	T2-12	56 − 20	72 − 6	64.3 %	91.7 %
8	7	F	T11,L1 HV(FS); L4 HV(SS)		38	29	T11,L1 HVR	L2 Y-shaped osteotomy	T10-L3	T8-L5	93 − 41	57 − 14	56.1 %	76.1 %
9	10	F	T8 HV(SS)with contralateral bar; *FS*:T5-9	7-10th fused rib with chest wall defect; Syringomyelia in thoracic spine	26	28	T8 Y-shaped osteotomy	T9 Y-shaped osteotomy	T6-10	T4-L1	68 − 28	45 − 22	58.8 %	51.1 %
10	2.5	M	T12 HV(FS); T11 WV		12	30	T12 HVR	T11 Y-shaped osteotomy	T10-L2	T8-L2	93 − 10	56 − 9	89.0 %	83.9 %
11	5	M	T6 HV(SS); T10 BFV, *FS*:T2-3,T4-5	3-6th fused rib; SCM: type I; Low conus:L3	88	24	T6 HVR	T9 Y-shaped osteotomy	T3-9	T4-L3	50 − 14	63 − 24	72.8 %	62.0 %

**Notes**:M, male; F, female;HV, hemivertebra; WV, wedge vertebra; BFV, butterfly vertebra; HVR, hemivertebra resection; T, thoracic; L, lumbar; FS, full segmented; SS, semi-segmented; SCM, spinal cord malformation; *FS*, failure of segmentation

### Initial surgery

The mean age at initial surgery was 6.6 ± 2.6 (2.5–10) years. The mean follow-up period was 36.1 ± 21.6 (11 to 88) months. The mean flexibility of the spine was 17.4 % (0.7–39.4 %). One stage posterior hemivertebrectomy or Y-shaped osteotomy with unilateral short fusion was performed (Fig. 2). The mean fusion level was 4.6 ± 1.3 (2 to 6) segments.

The mean coronal major curve was 67.4° (48 to 93) before operation, which improved to 23.7° (10 to 36) in postoperatively, with a mean correction of 64.8 %. The mean thoracic kyphosis was 48.0° (16 to 92) before operation and 29.3° (18 to 47) after operation. The mean lumbar lordosis was 55.5° (43 to 83) before operation and 40.3° (30 to 58) after operation. Alignment in the sagittal plane was either maintained or improved in all patients. The trunk shift, T1-12 and T1-S1 length was not significant difference between preoperative and postoperatively. The apex vertebra translation (AVT) was improved significantly before and after operation (*P* = 0.004) (Table 2).

**Table.2:** Radiographic data of patients in coronal and sagittal plane in initial surgery

	Preoperative	Postoperative	Improvement (%)	P value
Coronal plane				
Majorcurve Cobb angle (°)	67.4 ± 18.6	23.7 ± 9.9	64.8	*0.000
T1-12(cm)	14.7 ± 1.9	15.1 ± 2.9	-	0.535
T1-S1(cm)	25.1 ± 3.8	26.0 ± 5.2	-	0.463
TS(cm)	1.1 ± 1.2	0.7 ± 0.6	-	0.154
AVT(cm)	3.9 ± 2.1	1.6 ± 1.0	-	*0.004
Sagittal plane				
Thoracic kyphosis(°)	48.0 ± 26.4	29.3 ± 11.4	18.1	*0.034
Lumbar lordosis(°)	55.5 ± 13.5	40.3 ± 11.3	27.4	*0.011
SVA(cm)	-0.4 ± 2.9	0.9 ± 2.6	-	0.273

**Notes**: TS, trunk shift; AVT, apex vertebra translation; SVA, sagittal vertebral axis

* Means there is statistical significant difference

### Second surgery

**Fig. 3:**
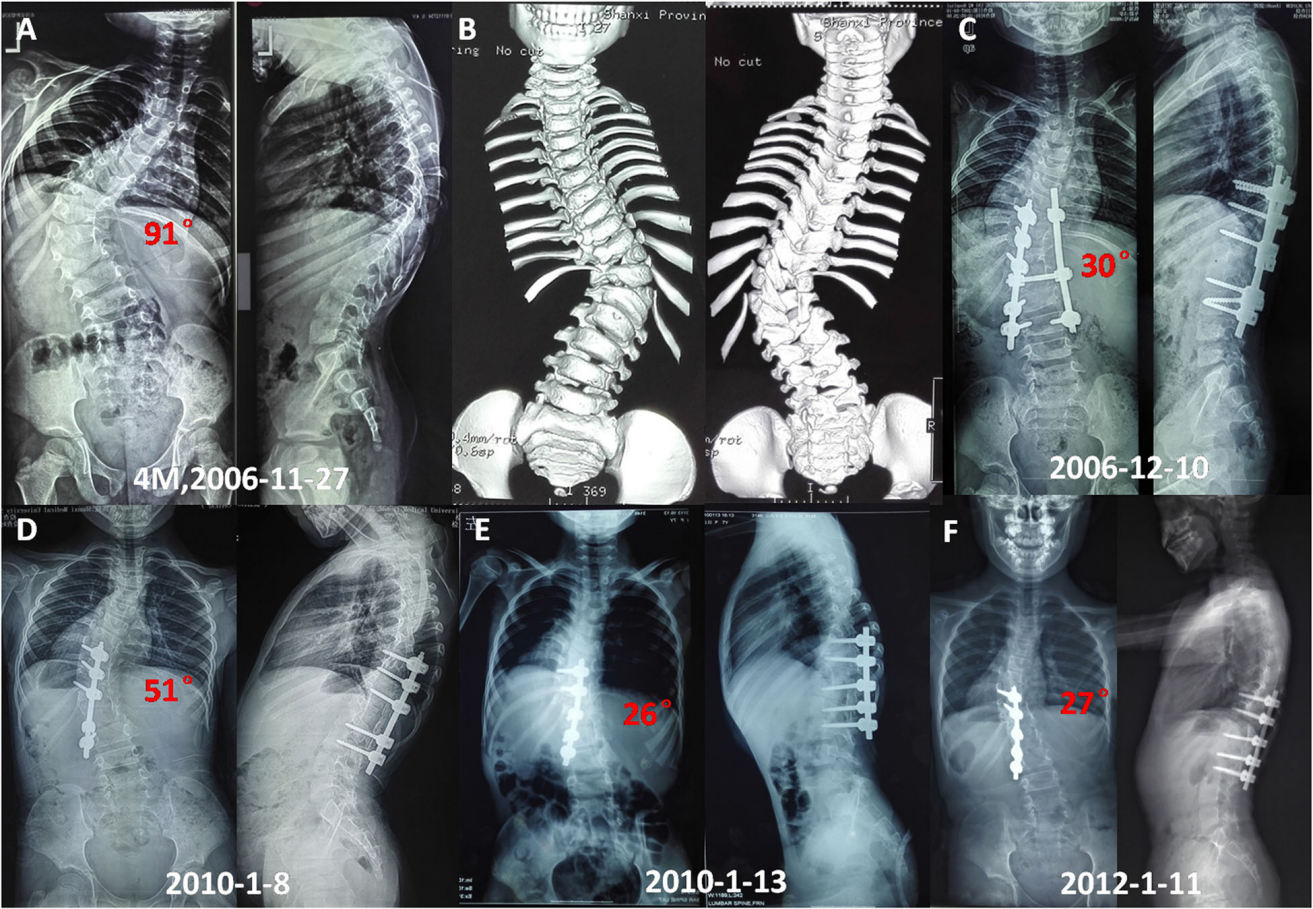
A 4 years old boy with complex CS. Radiographs and 3D CT showed T10, 12 full-segmented hemivertebra with concave contralateral bar, and 9th bifid rib (**A,B**). He underwent T10 and T12 hemivertebrectomy at initial surgery with short fusion(**C**). Second T11 Y-shaped osteotomy surgery was performed after 36 months because curve progression. Radiographs images obtained preoperatively, postoperatively and at the latest follow-up 24 months later (**D,E,F**)

**Fig. 4:**
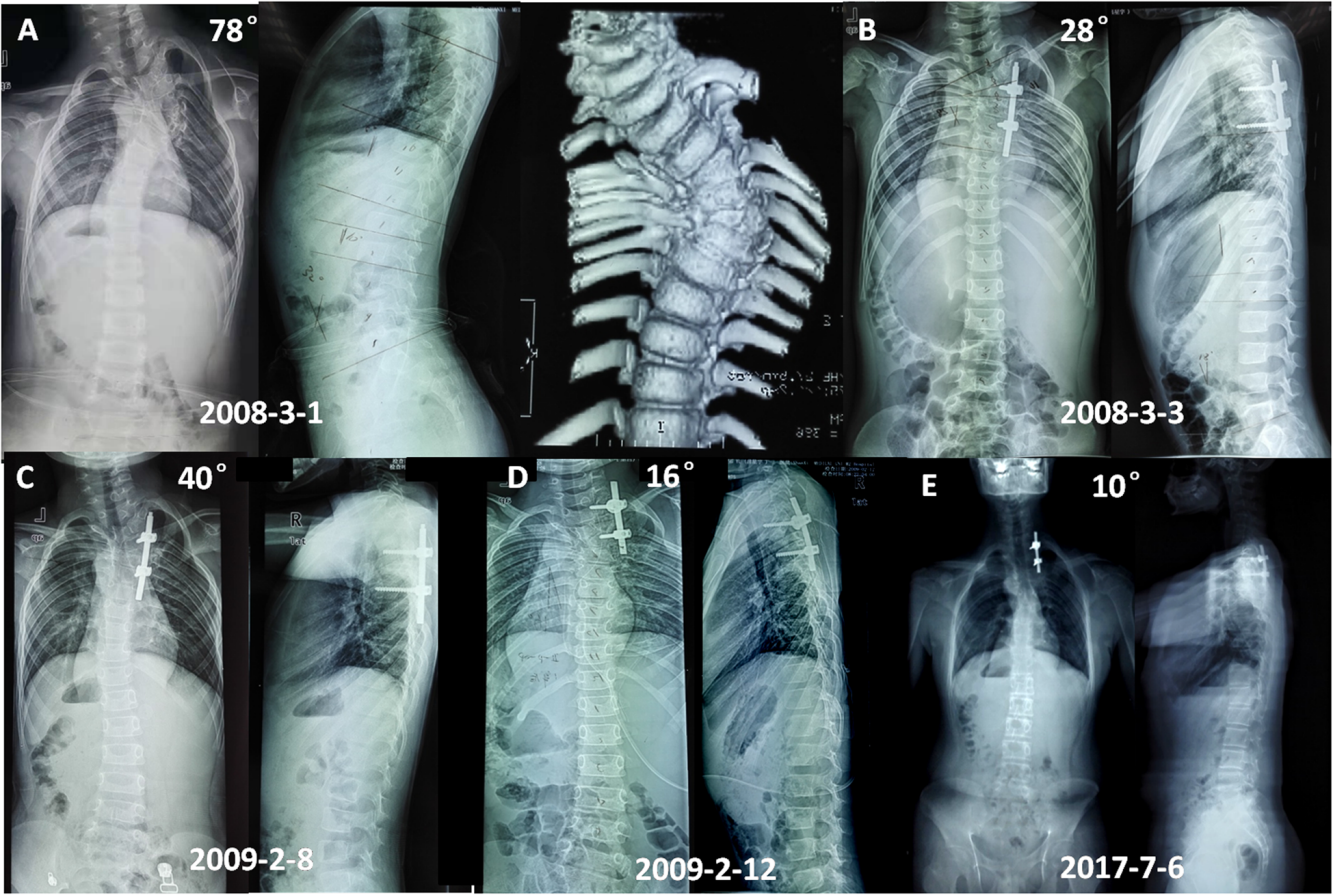
An 8 years old boy with complex congenital scoliosis. Radiographs and 3D CT showed T5 semi-segmented hemivertebra, T2-4 bloc vertebra and concave fused ribs (**A**). He underwent T5 HVR with unilateral short fusion at initial surgery (**B**). Second surgery including T2 Y-shaped osteotomy was performed after 11 months because proximal curve progression. Radiographs images obtained preoperatively, postoperatively and at the latest follow-up 101 months later (**C,D,E**)

In all, the mean follow-up period was 72.5 ± 23.8 (42 to 112) months. The mean follow-up period after second surgery was 36.5 ± 23.5 (18 to 101) months. The mean flexibility of the spine was 17.8 % (8–33.3 %). Y-shaped osteotomy in apex point with unilateral or bilateral fusion was performed in ten patients (Figs. 3 and 4). The mean fusion level was 7.5 ± 2.4 segments (range, 3–11 segments).

The mean coronal major curve was 50.3° (40 to 72) before operation, which improved to 15.5° (6 to 26) postoperatively and 17.4° (10 to 39) at the latest follow-up, with a mean correction of 69.2 and 65.4 %. The mean thoracic kyphosis was 44.6° (30 to 83) before operation and 34.1° (20 to 57) at the latest follow up. The mean lumbar lordosis was 51.3° (35 to 72) before operation and 47.2° (34 to 58) at the final follow up. The T1-12 length was 15.1 ± 2.9 cm after initial surgery, which increase to 18.6 ± 1.9 cm at the final follow up. The average growth of T1-12 is 0.58 cm per year. The T1-S1 length was 26.0 ± 5.2 cm after initial surgery, which increase to 31.6 ± 3.6 cm at the final follow up. The average increase of T1-S1 is 0.93 cm per year. The apex vertebra translation (AVT) was improved significantly before and after operation (*P* = 0.001), and kept stable at the final follow up (Table 3).

**Table.3:** Radiographic data of patients in coronal and sagittal plane in second surgery

	Preoperative	Postoperative	Improvement (%)	P value	Final follow-up	Improvement (%)	P value
Coronal plane							
Majorcurve Cobb angle (°)	50.3 ± 10.0	15.5 ± 6.5	69.2	*0.000	17.4 ± 8.6	65.4	0.418
T1-12(mm)	16.2 ± 2.2	16.0 ± 1.5	-	0.806	18.6 ± 1.9	-	**0.002
T1-S1(mm)	27.9 ± 3.5	28.1 ± 3.5	-	0.781	31.6 ± 3.6	-	**0.026
TS(mm)	1.0 ± 1.2	1.1 ± 0.7	-	0.747	1.4 ± 1.0	-	0.525
AVT(mm)	2.9 ± 1.0	1.2 ± 0.7	-	*0.001	1.4 ± 0.9	50.0	0.309
Sagittal plane							
Thoracic kyphosis(°)	44.6 ± 18.0	34.2 ± 6.9	23.3	0.093	34.1 ± 12.8	23.5	0.966
Lumbar lordosis(°)	51.3 ± 11.7	40.2 ± 4.9	21.6	*0.007	47.2 ± 12.2	7.9	0.095
SVA(mm)	-1.5 ± 3.1	0.1 ± 3.0	-	0.216	-1.1 ± 3.8	-	0.525

**Notes**: TS, trunk shift; AVT, apex vertebra translation; SVA, sagittal vertebral axis.

Statistical significant difference: * Pre-OP *versus* Post-OP; ** Post-OP *versus* Follow Up

### Complications

None of the patients underwent intra-operative complication. There were no neurologic complications. No infection, implant failures, pseudoarthrosis and significant correction loss was noted during the follow-up.

## Discussion

Congenital spinal deformity is always an early-onset scoliosis because of its embryological origin [13]. Some complex congenital scoliosis were caused by failure of segmentation in concavity of multiply vertebrae, hemivertebra combined with contralateral unsegmented bar, or vertebra anomalies with concave fused rib. Most of them are severe, rigid and aggravating quickly, producing enormous asymmetric growth. The osteotomy was always needed at the apex of sharp scoliosis or kyphosis [14]. Definitive posterior spinal fusion was not suitable because of it may result in a short trunk and pulmonary dysfunction. Implant failures of standard GR technique for these deformities remains the biggest challenge [15].Hybrid technique of posterior osteotomy with short fusion and GR distraction was reported and good preliminary clinical outcomes were achieved in previous studies [16, 17]. However, repeated lengthening procedures resulted in high complications and psychological disorder. In our series, 11 selected patients presenting complex congenital scoliosis were treated with two-staged posterior osteotomy surgery with short fusion.

The concept of this technique is to secure the growth of the patient’s spinal length and to avoid multiple operations while allowing some amount of the occurrence of crankshaft phenomenon. Previous study suggested that incidence of crankshaft was more frequent the earlier the surgery and in curves greater than 50°. But the number of discs in the fused segments and the length of the fusion were not found to be of significance [18]. The object of initial surgery is to correct partly and stabilize the spine in the optimum position by means of osteotomy in apex vertebra region. Other than avoiding deteriorating more severe or unbalanced curves, it can minimize any possible adverse effects on lung growth if the curvature is in the thoracic region. All curves must be carefully monitored to skeletal maturity after initial surgery. Some further curve progression may necessitate additional surgery especially during the adolescent growth spurt. If the curve progression in coronal and sagittal was significant, or severe PJK emerged, second osteotomy or extending the spinal fusion may be necessary to obtain a balance spine. In our case series, the mean fusion level was 4.6 segments in initial surgery. After average three years, second surgery was performed to further correct the residual or de novo curve and realign the spine.

Wang et al. reported their preliminary results of hybrid technique of 1-stage posterior osteotomy with short segmental fusion and dual growing rods for the treatment of 7 patients with congenital scoliosis [16]. The mean scoliosis improved from 81.4° to 40.1° after initial surgery and was 41.0° at the last follow-up or final fusion. The increase of T1-S1 length is 1.23 cm per year. Sun et al. also reported their results of 13 patients diagnosed with long-spanned CS treated with hybrid growing rods technique [17]. There were 8 patients with single rod while 5 with dual rods. The mean postoperative follow-up was 41.6 months. The major curve improved from 86.5° to 12.0° after initial surgery. The increase of T1-S1 length was 1.31 cm per year during the follow-up. Two complications were identified in two patients, including one with rod fracture and one with proximal junctional kyphosis.

Compare to their case series, we treated these complex congenital scoliosis using two staged osteotomy technology. The mean major curve improved from 67.4° to 23.7° after initial surgery and was 17.4° at the final follow-up. The increase of T1-S1 length is 0.93 cm per year, which is a bit lower than their studies. In our opinion, two factors contribute to this difference. On the one hand, second osteotomy shortening spine length. On the other hand, failure of segmentation in multiply vertebrae among of these cases restricted the growth of spine. Uehara M, et al. described the clinical outcomes of 2 patients with syndromic EOS who received 2-stage posterior spinal fusion [19]. Major curve Cobb angle improved from 100 to 77 degrees to 46 and 48 degrees at the final follow-up, respectively. Thoracic height improved from 16.0 to 14.8 cm before surgery to 20.6 and 21.1 cm at final follow-up, respectively. In our cases, the average height of T1-12 from 15.1 cm improved to 18.6 cm at the latest follow up at mean age of 12.6 years.

Excision of hemivertebra or Y-shaped osteotomy is attractive as a powerful procedure because it removes the primary cause of the scoliosis, which could eliminate driving force of asymmetric growth potential around apex and maintain spinal longitudinal growth. A closing wedge or Y-shaped osteotomy can shorten the spine and correct spinal deformity. In addition, shortening of the vertebral column is likely to avoid traction on the spinal cord and neurological complications during corrective surgery [20]. In present study, five patients had intraspinal anomalies, including 2 split spinal cord malformations, presenting as both bony and fiber septum, 1 synringomyelia and 3 tethered cord. Prophylactic neurosurgical intervention was not performed before spinal corrective surgery. Luckily, none of them had perioperative neurological complications. Shen et al. also suggested that patients with congenital scoliosis associated with SCM can safely and effectively undergo spinal deformity correction, and prophylactic detethering prior to scoliosis surgery may not be necessary [21].

For unilateral fusion, there was previous report in congenital hemivertebra and satisfactory outcomes were obtained [11]. Of much greater importance is that tethering growth on the convex side of the curve can lead to spontaneous correction of the curvature due to continued growth on the concavity. Therefore, care is taken not to strip the paraspinal muscles on the concavity of the curve so as not to interfere with the growth potential on this side of the spine [15]. Of course, it will be not that effective as a corrective procedure in patients with a unilateral unsegmented bar because there is less concave growth potential.

Several limitations of this study should be addressed. First, the strength of the results was limited by relative small sample size. Second, the inherent risk of data inaccuracy was present due to retrospective nature of the study. Third, this study lacked a control group of patients who underwent hybrid osteotomy and GRs technology. Finally, considering to some patients are very young and far from mature bone, the long follow-up was needed in the future.

## Conclusions

This study retrospectively evaluated 11 young children with complex CS who had undergone two staged osteotomy treatment. Good clinical and radiological outcomes can be achieved without severe complications. This procedure can be an option of treatment for complex congenital scoliosis.

## Data Availability

The datasets used and/or analyzed during the current study are available from the corresponding author upon reasonable request.

## Abbreviations

CS: Congenital scoliosis
HV: Hemivertebra
GRs: Growing Rods
EOS: Early onset scoliosis
SCM: Spinal cord malformation
CT: Computer tomography
MRI: Magnetic resonance imaging
TS: Trunk shift
SVA: Sagittal vertical axis
SPSS: Statistic Package for Social Science
AVT: Apex vertebra translation
VEPTER: Vertical expandable prosthetic titanium rib
